# Transformation from Self-Focusing to Self-Defocusing of Silver Nanoparticles

**DOI:** 10.3390/nano11102485

**Published:** 2021-09-24

**Authors:** Jijuan Jiang, Yang Jia, Tong Wu, Yachen Gao

**Affiliations:** 1 College of Electronic Engineering, Heilongjiang University, Harbin 150080, China; 02781@qqhru.edu.cn (J.J.); 1192153@s.hlju.edu.cn (Y.J.); 124wutong@163.com (T.W.); 2 College of Communication and Electronic Engineering, Qiqihar University, Qiqihar 161000, China

**Keywords:** silver nanoparticles, third-order nonlinear refraction, fifth-order nonlinear refraction, Z-scan

## Abstract

The nonlinear refraction of silver nanoparticles (AgNPs) in n-hexane was studied by using the closed-aperture Z-scan technique with a 532 nm nanosecond laser. It was found that, the nonlinear refraction of AgNPs shows the coexistence and transformation from self-focusing to self-defocusing. Specifically, self-focusing occurs at low excitation intensity, self-defocusing occurs at high excitation intensity, and coexistence of self-focusing and self-defocusing occurs at relatively moderate excitation intensity. The experimental results were analysed and discussed in terms of third-order and fifth-order nonlinear refractive effect. Specifically, the self-focusing is caused by the positive third-order nonlinear refraction, the self-defocusing is induced by the negative fifth-order nonlinear refraction, and the transformation from the self-focusing to self-defocusing at medium excitation intensity is caused by the competition of third-order and fifth-order nonlinear refraction. Finally, the third-order refractive index and fifth-order refractive index were obtained.

## 1. Introduction

The nonlinear optical (NLO) properties of nanomaterials have been extensively researched due to their applications in the fields of photothermal effect [1], optical sensing [2], photocatalysis [3]. Nonlinear refraction is an important branch of NLO properties. The effects of self-focusing, self-defocusing, self-phase modulation (SPM), optical phase conjugation (OPC) and optical bistability caused by nonlinear refraction have broad application prospects in the field of optical limiting [4], optical switching [5], spatial optical soliton transmission [6] and so on.

The NLO effect of noble metal nanoparticles is greatly enhanced due to their unique localized surface plasmon resonance (LSPR) behaviour [7,8,9,10]. The nonlinear refractive properties of gold, silver and other noble metal nanoparticles have been widely studied [10,11,12,13,14,15,16,17,18,19,20]. It has been reported that the nonlinear refractive index of composite materials with noble metal nanoparticles is greatly enhanced due to the effect of noble metal nanoparticles [9,10]. For example, Li et al. have reported that the third-order nonlinear refractive index of silver doped Nd: YAG single crystal is four times of magnitude higher than that of non-implanted Nd: YAG (without Ag NPs) [10]. Fu et al. studied the optical nonlinearity of pure CdS nanoparticles and Ag@CdS, and they believe that the enhancement of the local field caused by the strong SPR absorption of the cubic silver core enhance the nonlinear response of CdS nanoparticles [9]. The relationship between the third-order and the higher-order susceptibility obtained by Falcão-Filho et al. through theoretical research demonstrates that the increase of the third-order polarizability will inevitably induce the height of other higher-order polarizability, which will lead to the coexistence and competition between the high-order refractive index and the low-order refractive index [21]. It can also be predicted that the high-order and low-order refractive index with opposite symbols will induce self-focusing and self-defocusing; in fact, for the third- and fifth-order refractive index, it has been spotted in other materials [11,20,22,23,24]. For example, research conducted by Talita et al. on the gold nanorods shows that fifth-order nonlinearity also contributes to refraction. Their experiments have observed the coexistence and transformation of third- and fifth-order refraction with opposite signs [11]. Ganeev et al. studied the third- and fifth-order nonlinear refraction of pseudo isocyanide (PIC) dye aqueous solution [22]. They found that the PIC nonlinear refraction presents a double peak and double valley configuration at high laser intensity, and they attribute this phenomenon to the action of fifth-order nonlinearity. However, this transition was not seen in the AgNPs experiment of Falcão-Filho. As far as we know, there are few reports about the transformation and coexistence of self-focusing and self-defocusing induced by the opposite sign of the high-order and low-order refractive index for Ag NPs. Silver nanoparticles are often selected as dopants to improve the optical nonlinear properties of composites. Therefore, it is necessary to study the conversion of nonlinear refractive properties of AgNPs.

In this work, we research the nonlinear refractive properties of AgNPs in n-hexane using closed aperture Z-scan technique under different laser intensities. And we analyze and discuss the mechanism of nonlinear refraction based on third-order and fifth-order nonlinear refraction theory.

## 2. Experiment

The AgNPs (they were uniformly dispersed in n-hexane) used in the experiment were from Nanjing Xianfeng nanomaterials technology Co., Ltd. (Nanjing, China). The morphology of AgNPs was characterized by transmission electron microscopy (TEM). In order to obtain the linear absorption properties of the sample, a UV-2250 UV-Vis spectrophotometer (TU-1901, Persee, Auburn, CA, USA) was used to determine the linear absorption spectrum of the sample.

**Figure 1:**
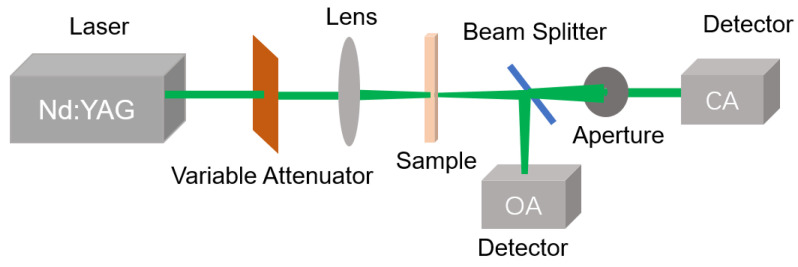
Experimental setup for the Z-scan measurements.

The nonlinear refraction of Ag NPs was investigated by using Z-scan technology. As shown in Figure 1, the Z-scan setup is similar to that described in the existing reports [11,25]. In Figure 1, an Nd: YAG laser system was used as the excitation source emitting 5 ns 532 nm laser with a repetition rate of 10 Hz. The pulsed laser beam has a lateral distribution and a time distribution approximate to the Gaussian distribution. The variable attenuator was used to control the energy of the excitation laser. The lens with a 10 cm focal length was used to focus the laser on the sample. The direction of beam propagation after the lens is specified as the z-axis. The sample is fixed on a translation table controlled by a computer program moving along the z-axis. Two detectors (OA and CA) were used to measure the energy of the pulsed laser transmitting through the sample. The former without apertures in front of the detector is used to measure nonlinear absorption, which is called open aperture (OA) Z-SCAN, while the CA detector with an aperture is applied to measure nonlinear refraction, which is called closed aperture (CA) Z-SCAN. In fact, in order to avoid the influence of nonlinear absorption, nonlinear refraction should be revealed from the transmittance of the CA detector divided by the OA one.

## 3. Results and Discussion

**Figure 2:**
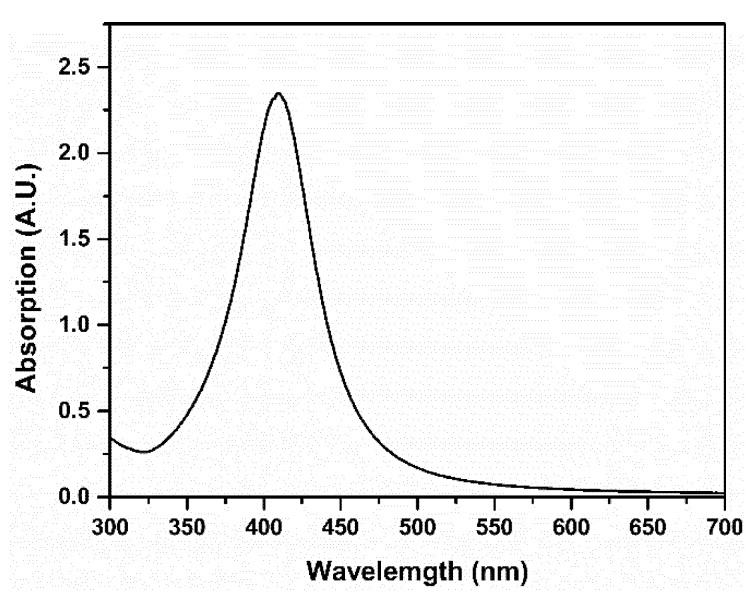
The linear absorption spectrum of AgNPs.

**Figure 3:**
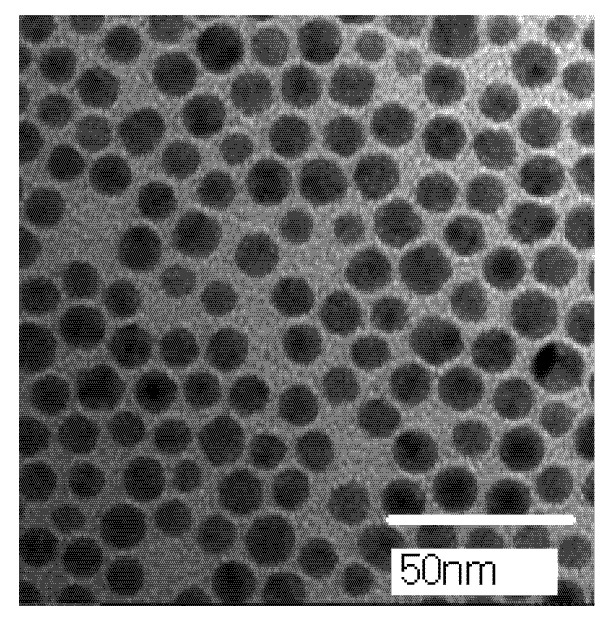
The TEM image of AgNPs.

The linear absorption and shape of nanoparticles will affect their optical nonlinear properties [26]. The UV-Vis absorption spectrum of AgNPs is shown in Figure 2. It can be seen from Figure 2 that AgNPs have a strong absorption band at about 410 nm, which can be attributed to the SPR of AgNPs [12,13,26]. The TEM image of AgNPs is shown in Figure 3 and it can be determined that the average diameter of AgNPs is about 10 nm.

**Figure 4:**
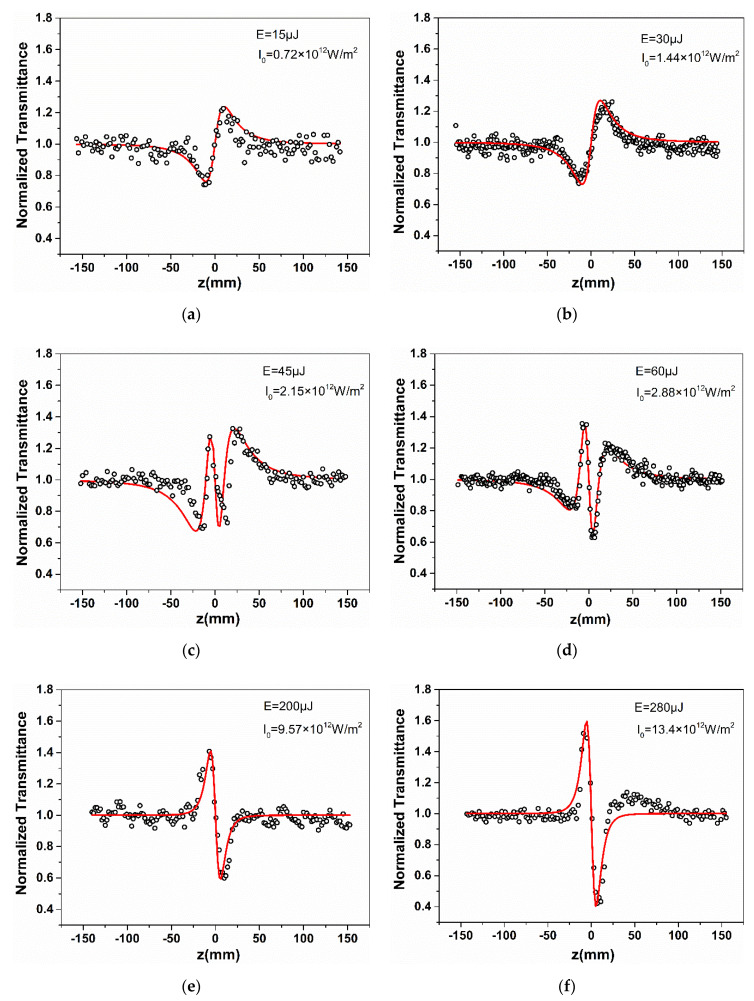
CA Z-scan curves of AgNPs under different laser energy. (**a**) laser energy of 15 μJ (irradiance at focus of 0.72 × 10^12^ W/m^2^); (**b**) laser energy of 30 μJ (irradiance at focus of 1.44 × 10^12^ W/m^2^), (**c**) laser energy of 45 μJ (irradiance at focus of 2.15 × 10^12^ W/m^2^); (**d**) laser energy of 60 μJ (irradiance at focus of 2.87 × 10^12^ W/m^2^); (**e**) laser energy of 200 μJ (irradiance at focus of 9.57 × 10^12^ W/m^2^); (**f**) laser energy of 280 μJ (irradiance at focus of 13.4 × 10^12^ W/m^2^). The small circles and the red solid curves are experimental data and theoretical fitting, respectively.

The Nd: YAG laser system was used in this Z-scan study. It provided a pulse width with 5 ns at 532 nm and repetition rate of 10 Hz. In our experiment, a thick quartz cell with 2 mm was used to hold AgNPs solution. The laser energy E is adjusted to 15, 30, 45, 60, 200, and 280 μJ (accordingly, the peak irradiance $I_{0}\;$ of the laser beam at the focusing position z = 0 is $0.72\times 10^{12}\;W/m^{2}$, $1.44\times 10^{12}\;W/m^{2}$, $2.15\times 10^{12}\;W/m^{2}$, $2.87\times 10^{12\;}W/m^{2}$, $9.57\times 10^{12}\;W/m^{2}$ and $13.4\times 10^{12}\;W/m^{2}$, respectively). The Z-scan results are shown in Figure 4, where the scattered points and red solid curves are experimental data and theoretical fitting (which will be introduced later), respectively.

From the results of the experiment in Figure 4 we find that the excitation intensity strongly affects the sign of the nonlinear refractive index. Firstly, in Figure 4a,b, the CA Z-scan curves have a valley before z = 0 and a peak after z = 0 at lower intensities, $0.72\times 10^{12}\;W/m^{2}$ corresponds to Figure 4a, and $1.44\times 10^{12}\;W/m^{2}$ corresponds to Figure 4b, which clearly shows that the nonlinear refractive index sign is positive self-focusing. When the intensity increases to $9.57\times 10^{12}\;W/m^{2}$, as shown in Figure 4e, the experimental results are completely opposite to those at low intensity, that is, the configuration of peak before valley, which indicates that the nonlinear refractive index sign is negative self-defocusing. The experimental result when the intensity is $13.4\times 10^{12}\;W/m^{2}$, is analogue to Figure 4e, as shown in Figure 4f. It is worth noting that the peaks and valleys are sharper and steeper than the self-focusing at high intensity, and the z-axis spacing of the peaks and valleys is closer. In addition, when AgNPs are irradiated with relatively moderate excitation intensities $2.15\times 10^{12}\;W/m^{2}$ and $2.87\times 10^{12}\;W/m^{2}$, the double peak valley shown in Figure 4c,d appears.

Now, we qualitatively analyse the nonlinear refraction effect of AgNPs. In the experiment, the laser wavelength is λ=532 nm, and the waist radius of the laser beam $\omega_{0}$ is about 43 μm, measured by using the knife-edge method. By $z_{0}={\pi \omega }_{0}/\lambda$ the Rayleigh diffraction length can be determined $z_{0}≅12\;\mathrm{mm}$, which satisfies the thin sample approximation condition $\left(z_{0}\gg L\right)$. The research in Refs. [21,25] both show that when high-order nonlinear refraction occurs, the peak valley z-axis transverse distance $Z_{p-v}$ of the normalized transmittance curve in the experimental curves will decrease [21,25]. Typical values have been calculated and reported, when the third-order nonlinear refraction occurs $Z_{p-v}\approx 1.7z_{0}$, and in the case of fifth order,$\;Z_{p-v}\approx 1.2z_{0}$ [25]. From the experimental curves, the case of medium excitation intensity is more complex. We first discuss the case of low intensity and high intensity. It is clear to see from the experimental data in Figure 4 that compared to low-intensity excitation (Figure 4a,b), under high-intensity excitation (Figure 4e,f) $Z_{p-v}$ is significantly reduced. It should be determined that the increase in intensity leads to the occurrence of higher-order nonlinear refraction. It is noticed that the transmittance peak valley difference $∆T_{p-v}$ does not change significantly when the laser intensity is $0.72\times 10^{12}\;W/m^{2}$ and $1.44\times 10^{12}\;W/m^{2}$. Fan et al. used femtosecond laser to obtain the nonlinear refraction curves of AgNPs at different laser intensities [13]. Their experiments show that the increase of laser intensity leads to the increase of peak valley difference $∆T_{p-v}$ of transmittance, and the ratio of $∆T_{p-v}$ to laser intensity I is basically unchanged. They attributed nonlinear refraction to third-order. In contrast, our experimental results show that the double enhancement of laser intensity I does not double that of $∆T_{p-v}$. Falcão-Filho et al. also reported a simple, effective and more general method to determine the functional relationship between $\left|{\Delta T}_{p-v}\right|/I$ and I by solving $\left|{\Delta T}_{p-v}\right|/I$ according to $∆T_{p-v}=0.406{\left(1-S\right)}^{0.25}k\gamma I_{0}\left(t\right)L_{\mathrm{eff}}$. They believe that if there is only a third-order contribution, the ratio $\left|{\Delta T}_{p-v}\right|/I$ should be constant [21]. Therefore, it is certain that the pure third-order nonlinear refraction does not occur at intensity $1.44\times 10^{12}\;W/m^{2}$ in our experiment. We consider that the contribution of the fifth-order refractive index with opposite signs can suppress the peak valley difference $∆T_{p-v}$ caused by the third-order nonlinear refraction. For the self-defocusing behavior at high excitation intensity, the peak valley transverse spacing $Z_{p-v}$ is obviously smaller than those at low excitation intensity. It can be inferred that the fifth-order nonlinearity is dominant, and the third-order component may be negligible. Therefore, the negative fifth order nonlinear refraction dominates the self-defocusing phenomenon of AgNPs at high intensity. For the experimental results shown in Figure 4c,d obtained at medium excitation intensity, certainly, we cannot explain it with a nonlinear refraction model containing only third-order. In the study of the nonlinear refraction of gold nanorods and PIC, the experimental results of double peaks and double valleys similar to those shown in Figure 4c,d were found, which were interpreted as the combined effect third-order and fifth-order refraction with opposite signs [11,22]. Therefore, from our experimental results, it clearly shows the positive and negative change process of the nonlinear refractive index sign, which indicates that the coexistence of self-focusing and self-defocusing occurs in the process of single Z-scan as the sample approaches the focus (z = 0).

It has been reported that different methods are used to obtain the nonlinear refractive index [21,25,27,28,29]. We refer to the theoretical reports on the third- and fifth-order nonlinear refractive index of thin media under the action of a Gaussian laser [21,25,27]. The expressions of the axial phase shift including the third- and fifth-order are as follows [21]:

(1) $$\Delta {\phi_{0}}^{\left(3\right)}\left(t\right)+\Delta {\phi_{0}}^{\left(5\right)}\left(t\right)=k\gamma I_{0}\left(t\right)\frac{1-e^{-\alpha_{0}L}}{\alpha_{0}}+k\eta I_{0}^{2}\left(t\right)\frac{1-e^{-2\alpha_{0}L}}{2\alpha_{0}}$$

where $\Delta {\phi_{0}}^{\left(3\right)}\left(t\right)$ and $\Delta {\phi_{0}}^{\left(5\right)}\left(t\right)$ represent the axial phase shift caused by the third-order nonlinear refraction and the fifth order nonlinear refraction, respectively, $I_{0}$ is the peak irradiance of the laser beam at the focusing position; γ is the third-order refractive index coefficient; η is the fifth order refractive index coefficient, L is the thickness of the sample; $\alpha_{0}$ is the linear absorption coefficient.

Under the condition of the first-order approximation, the normalized transmittance T(z) at the far-field aperture can be expressed as [21]:

(2) $$T\left(z\right)=1+\frac{4∆\phi_{0}^{\left(3\right)}\left(t\right)x}{\left(1+x^{2}\right)\left(9+x^{2}\right)}+\frac{8∆\phi_{0}^{\left(5\right)}\left(t\right)x}{{\left(1+x^{2}\right)}^{2}\left(25+x^{2}\right)}$$

where $\;x=z/z_{0}$ is the relative position of the sample.

Using Equation (2) to fit the experimental data, the third- and fifth- order nonlinear refractive index coefficients were obtained, as shown in Table 1.

According to the data in Table 1, it can be seen that third-order and fifth-order are opposite from the perspective of the sign of the refractive index. From the perspective of numerical change, the third-order refractive index decreases, and the fifth-order refractive index increases with the increase in laser intensity. These indicate that there is competition between the third- and fifth- order. When the third-order nonlinear refraction plays a major role, it shows self-focusing property. On the contrary, when the fifth order nonlinear refraction plays a major role, the self-defocusing property is realized. When the third- and fifth-order effects are comparable, the self-focusing and self-defocusing coexist. Falcão-Filho et al. studied the high-order nonlinear refraction of AgNPs using a picosecond laser at 532 nm with different intensities in the range of $0.1\sim 1.5\;\mathrm{GW}/{\mathrm{cm}}^{2}$ [21]. The fifth order nonlinear refractive index obtained by them is about $2.7\times 10^{-31}\;m^{4}/W^{2}$, which is about an order of magnitude lower than that obtained by us. It is worth noting that in their experimental results, the fifth order effect is not enough to change the peak valley configuration of the closed aperture Z-scan. With the increase in laser intensity, it always shows self-defocusing behaviour. In our experiment, the fifth-order nonlinear refraction plays a major role, which leads to the transformation from self-focusing to self-defocusing in the CA Z-scan experiment. Talita et al. studied the high-order nonlinear refraction of gold nanorods excited by picosecond laser [11]. They found that the high-order nonlinearity is attributed to the high-order electronic states, and the generation of high-order can be regulated by adjusting the aspect ratio of gold nanorods. These studies have practical significance for application. They observed the CA Z-scan experimental results with double peaks-valleys and obtained the magnitude with $10^{-14}\;{\mathrm{cm}}^{2}/W$ of third-order refractive index and the magnitude with $10^{-23}{\mathrm{cm}}^{4}/W^{2}$ of fifth-order nonlinear refractive index, which is close to our results.

Finally, we try to explain the mechanism of nonlinear refraction. There are two kinds of effects that can explain the self-focusing and self-defocusing effects of nonlinear refractive index, namely electronic mechanism and thermal effect [30]. The thermal effect usually mentioned in solution is a slow accumulation process. For aqueous solution, the accumulation time of thermal effect is about 30 ns [12]. Although the solvent in our experiment is n-hexane, it can be considered that the cumulative time of thermal effect should be close to the duration of 5 ns laser pulse. In addition, in our Z-scan experiment, a repetition rate of 10 Hz can reduce the accumulation of thermal effects at lower intensities. According to the model reported by Hamanaka et al., the generation of hot electrons leads to nonlinear refraction [31]. The excitation laser with wavelength far from SPR peak may induce self-focusing for silver nanoparticles. The self-focusing at low excitation intensity in our experiment is consistent with their theory. Therefore, we believe that the self-focusing of silver nanoparticles at low intensity in our experiment originates from the generation of hot electrons in the conduction band, but the thermal contribution cannot be completely excluded. However, with the increase in sample temperature at high excitation intensity, the thermal accumulation effect may be dominant, leading to self-defocusing.

## 4. Conclusions

In this paper, the nonlinear refraction effect of AgNPs under different excitation intensities was researched by using the CA Z-scan technique. With the increase in laser intensity, it is found that, the nonlinear refraction of AgNPs shows the coexistence and transformation from self-focusing to self-defocusing. Specifically, the third-order nonlinear refraction generally occurs under weak excitation, while the fifth-order nonlinear refraction occurs under strong excitation, while the coexistence of third-order self-focusing and fifth-order self-defocusing occurs under medium excitation. Theoretical calculation shows that the magnitudes of the third- and fifth-order refractive index are $10^{-17}m^{2}/W$ and $10^{-30}m^{4}/W^{2}$, respectively. The self-focusing at low excitation is attributed to the contribution of hot electrons, while the self-defocusing at strong excitation is considered to be the result of thermal accumulation.

## Figures and Tables

**Table 1:** Nonlinear refractive index of AgNPs.

*I*_0_ (×10^12^ *W*/*m*^2^)	0.72	1.44	2.15	2.87	9.57	13.40
*γ*(×10^−17^ *m*^2^/*W*)	10.18	8.06	13.20	6.72	→0	→0
*η*(×10^−30^ *m*^4^/*W*^2^)	—	−63.36	−204.01	−97.51	−4.11	−2.94
